# Distinct Synchronous Network Activity During the Second Postnatal Week of Medial Entorhinal Cortex Development

**DOI:** 10.3389/fncel.2020.00091

**Published:** 2020-04-21

**Authors:** Julia Dawitz, Tim Kroon, J. J. Johannes Hjorth, Huib D. Mansvelder, Rhiannon M. Meredith

**Affiliations:** Integrative Neurophysiology, Center for Neurogenomics and Cognitive Research, Vrije Universiteit Amsterdam, Amsterdam, Netherlands

**Keywords:** development, medial entorhinal cortex, giant depolarizing potentials, early network oscillations, GABA, synchronization

## Abstract

The medial entorhinal cortex (MEC) contains specialized cell types whose firing is tuned to aspects of an animal’s position and orientation in the environment, reflecting a neuronal representation of space. The spatially tuned firing properties of these cells quickly emerge during the third postnatal week of development in rodents. Spontaneous synchronized network activity (SSNA) has been shown to play a crucial role in the development of neuronal circuits prior to week 3. SSNA in MEC is well described in rodents during the first postnatal week, but there are little data about its development immediately prior to eye opening and spatial exploration. Furthermore, existing data lack single-cell resolution and are not integrated across layers. In this study, we addressed the question of whether the characteristics and underlying mechanisms of SSNA during the second postnatal week resemble that of the first week or whether distinct features emerge during this period. Using a combined calcium imaging and electrophysiology approach *in vitro*, we confirm that in mouse MEC during the second postnatal week, SSNA persists and in fact peaks, and is dependent on ionotropic glutamatergic signaling. However, SSNA differs from that observed during the first postnatal week in two ways: First, EC does not drive network activity in the hippocampus but only in neighboring neocortex (NeoC). Second, GABA does not drive network activity but influences it in a manner that is dependent both on age and receptor type. Therefore, we conclude that while there is a partial mechanistic overlap in SSNA between the first and second postnatal weeks, unique mechanistic features do emerge during the second week, suggestive of different or additional functions of MEC within the hippocampal-entorhinal circuitry with increasing maturation.

## Introduction

The medial entorhinal cortex (MEC) contains specialized cell types whose firing is tuned to aspects of an animal’s position and orientation in the environment, reflecting a neuronal representation of space (Moser and Moser, 2008). The spatially tuned firing properties of these cells quickly emerge during the third postnatal week of development in rodents (Langston et al., 2010; Wills and Cacucci, 2014). Firing patterns of grid cells, the most abundant spatially tuned cell-type in superficial MEC (sMEC) (Sargolini et al., 2006), depend on local inhibitory connectivity according to attractor network models (McNaughton et al., 2006; Couey et al., 2013; Pastoll et al., 2013). Recurrent inhibition in sMEC layers starts to develop during the third postnatal week (Couey et al., 2013; Pastoll et al., 2013). Additionally, it has recently been shown that proper maturation of the hippocampal circuits depends on layer II MEC stellate cells, starting from postnatal day (P)14 onward (Donato et al., 2017). Thus, during the third postnatal week, there is a rapid maturation of MEC circuits that drives the development of the hippocampal formation. However, MEC network maturation starts well before the third postnatal week.

In many brain regions, spontaneous synchronized network activity (SSNA) has been shown to be essential for several developmental processes underlying early network maturation (see Spitzer, 2006; Blankenship and Feller, 2009 for reviews). Such SSNA has also been observed in the MEC during the first two postnatal weeks (Jones and Heinemann, 1989; Sheroziya et al., 2009; Namiki et al., 2013). During the first postnatal week, SSNA is initialized in layer III of the lateral entorhinal cortex (LEC), and then travels to MEC, hippocampus, and neocortex (NeoC; Namiki et al., 2013). Thus, SSNA of EC is synchronized with neocortical, CA1, and CA3 activity and has therefore been proposed as a cortical “pacemaker” whose intrinsic activity drives neighboring neocortical regions during early postnatal development (Garaschuk et al., 2000; Namiki et al., 2013). Indeed, it has been shown *in vivo* that during the first postnatal week MEC drives hippocampal sharp-waves (Valeeva et al., 2019). SSNA in EC depends on ionotropic glutamate receptors (iGluRs) (Jones and Heinemann, 1989; Sheroziya et al., 2009; Namiki et al., 2013) and it is suggested that the disappearance of SSNA in hippocampus and NeoC is mediated by the maturation of GABA-A receptor activity (Garaschuk et al., 2000; Allène et al., 2008; for review: Blankenship and Feller, 2009). Although SSNA is most extensively studied during the first postnatal week, field potential recordings have shown that in layer III of the MEC, SSNA peaks around P9/10 (Sheroziya et al., 2009). However, these data lack single cell resolution. Additionally, very little is known about the effects of GABAergic signaling on SSNA in MEC during the second postnatal week. In summary, while SSNA in MEC during the first postnatal week is well described, there are little data about the second postnatal week, while existing data lack single-cell resolution.

Therefore, the question arises whether SSNA of MEC during the second postnatal week is an extension of the activity observed during the first week or whether it has its own characteristics and mechanisms and therefore a potentially distinct function at cellular and computational levels. We hypothesize that MEC activity during the second postnatal week is in a transitional phase with some similarities to those reported during the first postnatal week but distinct emerging characteristics and mechanisms that could potentially prepare the layer II stellate cells for their determining role in hippocampal development from the third week onward.

To address this, we used calcium imaging and electrophysiology to dissect characteristics and mechanisms of MEC neuron activity during the second postnatal week. Upon mapping the SSNA during the second postnatal week, we determined the correlation of activation within MEC and between MEC and NeoC, CA1, and CA3. Finally, we probed the involvement of iGluRs, as well as GABA-A and GABA-B receptors during these developmental activity patterns. We find that while there is a partial mechanistic overlap in SSNA between the first and second postnatal weeks, unique mechanistic features do emerge during the second week, suggestive of different or additional functions of MEC within the hippocampal-entorhinal circuitry with increasing maturation.

## Materials and Methods

### Animal Usage

All procedures involving animals were conducted in accordance to Dutch regulations and were approved by the animal ethics committee (DEC) of the Vrije Universiteit Amsterdam. C57BL/6 mouse pups of both sexes aged from postnatal day 7 (P7) to P15 were used for slice experiments.

### Reagents

If not indicated otherwise, all reagents were purchased from Sigma–Aldrich. For pharmacology, the following concentrations of blockers were added to the standard artificial cerebral spinal fluid (ACSF): DL-2-amino-5-phosphonopentanoic acid (DL-APV, Abcam): 100 μM, 6-cyano-7-nitroquinoxaline-2,3-dione (CNQX, Abcam): 2 μM, Gabazine (Tocris): 10 μM (blockade of phasic and tonic), CGP 55845 (CGP, Tocris): 4 μM.

### Preparation of Horizontal Brain Slices

Horizontal entorhinal cortex slices were prepared as described previously (Dawitz et al., 2011). Briefly, animals were rapidly decapitated, and their brains dissected out in ice cold cutting solution containing (in mM): 110 Choline chloride, 26 NaHCO_3_, 10 D-glucose, 11.6 sodium ascorbate, 7 MgCl_2_, 3.1 sodium pyruvate, 2.5 KCl, 1.25 NaH_2_PO_4_ (Merck), and 0.5 CaCl_2_. 300 μm thick slices were obtained using a HR2 slicer (Slicer HR-2; Sigmann Elektronik, Hueffenhardt, Germany; vibration frequency: 36 Hz, vibration amplitude: 0.7 mm, propagation speed: 0.05 mm/s). After a minimum recovery period of 1 h, slices were transferred into a holding chamber containing ACSF with slightly elevated magnesium levels at room temperature composed of (mM): 125 NaCl, 26 NaHCO_3_, 10 D-glucose, 3 KCl, 2.5 MgCl_2_, 1.6 CaCl_2_, and 1.25 NaH_2_PO_4_ (Merck) and continuously bubbled with carbogen gas (95% O_2_, 5% CO_2_).

### Fura2-AM Bulk Loading and Two-Photon Data Acquisition

After recovery slices were transferred into an interface-chamber filled with 1 ml elevated magnesium ACSF heated to approximately 34°C. 50 μg Fura2-AM (Invitrogen) diluted in 9 μl DMSO and 1 μl Pluronic^®^ F-127 (20% solution in DMSO; Invitrogen) was pipetted directly on top of the entorhinal cortex and incubated from 20 min—for slices of P7/8 animals, to 40 min—for slices of P14/15. Slices from animals older than P12 were pre-incubated for three minutes in 3 ml ACSF with 8 μl 0.5% cremophor (Fluka) diluted in DMSO, to facilitate Fura2-AM uptake. After incubation slices were briefly transferred back into the holding chamber and any residual surface dye was washed off. To improve stability of recordings a poly(ethyleneimine)-solution [1 ml poly(ethyleneimine) in 250 ml boric buffer containing 40 mM boric acid and 10 mM sodium tetraborate decahydrate] was used to attach slices onto the recording chambers. After coating of the recording chambers for 1 h, slices were mounted in elevated magnesium ACSF. The attached slices were placed into a humidified interface container, perfused with carbogen and left for at least 1 h to (a) achieve a stable attachment and (b) to allow for esterase activity to take place within the neurons to trap and activate Fura2-AM. Full methodology, details of slice preparation and imaging are published previously (Dawitz et al., 2011).

Functional multiphoton calcium dye network imaging data were acquired on a Trimscope (LaVision Biotec) connected to an Olympus microscope using a Ti-sapphire (Coherent) laser tuned to 820 nm wavelength. All recordings of sMEC were acquired using a 20x lens (NA 0.95) and a 350 × 350 μm field of view. Parallel recordings of deep MEC (dMEC) and sMEC were obtained with a 10x objective (NA 0.3) and a 700 × 700 μm field of view. During data acquisition, slices were continuously perfused with oxygenated standard ACSF (recipe as above but with 1.5 mM MgCl_2_) heated to approximately 27°C. Superficial layers of the MEC were visually identified using light microscopy. Using a Hamamatsu C9100 EM-CCD camera as a detector, two time-lapse movies (2000 frames each) in sMEC or sMEC and dMEC were acquired with a sampling frequency of approximately 10 Hz. For neuron detection, at the end of each recording condition a z-stack ± 20 μm around the central plane with 1 μm optical slice thickness was acquired.

For simultaneous network imaging and cell-attached stellate cell recording (for solutions used, see section “Field Recordings and Analysis”), data were acquired on a Leica RS2 two-photon laser-scanning microscope tuned to 820 nm wavelength with a 20x lens and 400 × 400 μm field of view. Time-lapse data were acquired on a PMT at a rate of 565 ms/frame (425 frames each) in sMEC with simultaneous cell-attached recording. Spike rates were acquired using Clampex 10.2 (MDS Analytical Technologies) at an acquisition rate of 10 kHz. Cells in layer II were filled with Alexa-594 (80 μM, Invitrogen) for putative anatomical identification based on their morphology (Canto and Witter, 2012). Neuronal morphology was reconstructed using NeuroMantic software (Myatt et al., 2012).

Drugs were washed in for minimally 10 min prior to recording. Hippocampal and NeoC lesions were prepared under a low magnification microscope (4x) using a surgical knife. After lesioning, two time-lapse movies and a z-stack were recorded as described above.

### Analysis of Imaging Data

Custom-built Matlab^®^ (Mathworks) software was used to analyze calcium data (Hjorth et al., 2016). Neurons were detected within the z-stack recorded at the end of each condition. After localizing putative neuronal centers using local intensity peaks, deformed spheres were placed around these centers within the 3D stack to fit the putative neurons. A neuron was added to the contour mask if the spheres had the volume corresponding to a radius of 2–20 μm and the intersection of the sphere and the imaging plane had a minimum area of 25 μm^2^. By marking the pia, the distance for individual neurons in the slice to it could be calculated. The pia was indicated on the final mask to determine the distance to pia of individual neurons in the slice. For each detected neuron, the corresponding fluorescent raw trace was extracted from the two consecutive frames in time-lapse recordings and the relative fluorescence trace was calculated (ΔF/F). The baseline was estimated using the running median of the relative trace. Frames with a drop in intensity of at least 10% in the relative trace were considered as a putative event. Additionally, the event had to be at least 1 standard deviation (SD) below the baseline, and the trace had to remain significantly below this baseline for a minimum of five frames (tested with a one-sided *t*-test). To confirm onset times and to increase sensitivity of onset detection, we repeated this procedure three times excluding the detected putative events from the running median and using a two-sided *t*-test comparing the putative event frames against the filtered baseline for significance in the consecutive iterations. The result was manually inspected and corrected where necessary.

Groups of synchronized neurons were detected as follows: Onset times of detected events from all neurons were summed together on a frame-by-frame basis and the resulting activity vector was smoothed using a Gaussian with a full width at half-maximum of five frames (500 ms). All local peaks exceeding the threshold were defined as network events. The threshold was five times the SD of 500 activity vectors derived from the same traces but with randomly shuffled inter-event intervals. A neuron was assigned to the synchronized group if it participated in at least 40% of network events. during the trace.

**FIGURE 1:**
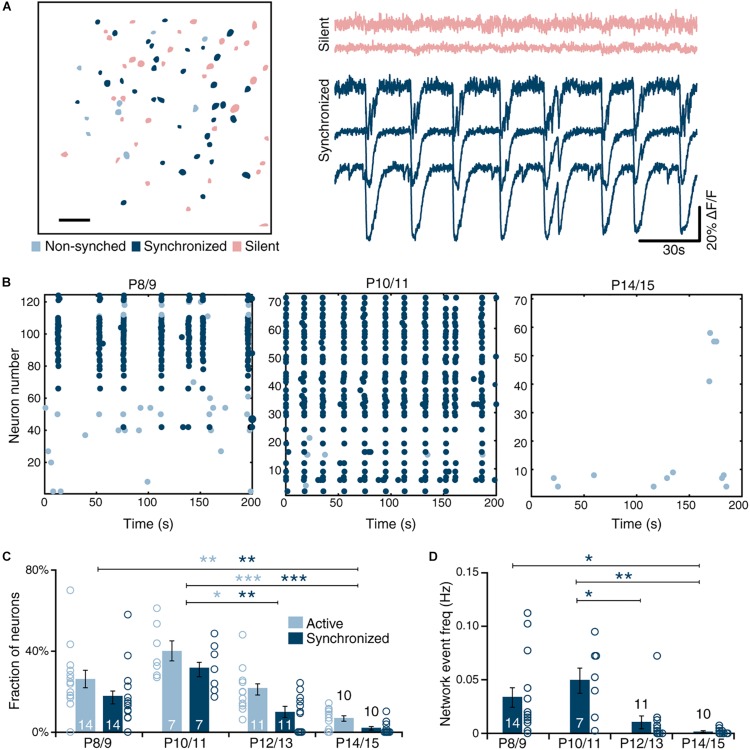
Developmental dynamics of spontaneous sMEC activity. **(A)** Left: Contour map of Fura2-AM ester bulk-loaded cells in superficial medial entorhinal cortex networks imaged at P10/11. Active neurons (light blue), silent neurons (red), and synchronized neurons (dark blue) indicated. Scale bar 50 μm. Right: Example traces of silent and synchronously active neurons. **(B)** Representative raster plots of superficial MEC (sMEC) network activity at P8/9, P10/11, and P14/15, color-coded as in **(A)**. **(C)** Quantification of the fraction of active and synchronized neurons during postnatal development showed a significant age-dependent peak at P10/11 in fraction of cells that are active [one-way ANOVA, *F*(3,41) = 10.1, *p* < 0.001; Bonferroni corrected, P8/9 vs. P14/15, *p* = 0.004; P10/11 vs. P12/13, *p* = 0.028; P10/11 vs. P14/15, *p* < 0.001], as well as the fraction of cells that are synchronized [one-way ANOVA, *F*(3,41) = 11.1, *p* < 0.001; Bonferroni corrected, P8/9 vs. P14/15, *p* = 0.008; P10/11 vs. P12/13 *p* = 0.001; P10/11 vs. P14/15, *p* < 0.001]. **(D)** Frequency of network events (see section “Materials and Methods” for definition) is highest at P10/11 and virtually absent by P14/15 [one-way ANOVA, *F*(3,41) = 5.8, *p* = 0.002; Bonferroni corrected: P8/9 vs. P14/15, *p* = 0.043; P10/11 vs. P12/13, *p* = 0.032; P10/11 vs. P14/15, *p* = 0.006]. Bar graphs represent the mean ± standard error of the mean (SEM). *N* shown in bars as the number of slices. Number of animals used, P8/9: 4, P10/11: 2, P12/13: 3. P14/15: 2. *p < 0.05; **p < 0.01; ***p < 0.001.

Thus, from this analysis, three categories of neurons were derived: silent neurons that do not show any activity, active neurons that show activity, and synchronized neurons that are active and whose activity is synchronized to other neurons in the network (Figure 1A). The frequency of each group and the network event frequency were calculated. Finally, another Matlab^®^ (Mathworks) script was used to align masks of different experimental conditions to extract repeated measurements of parameters for testing based on individual neurons. All frequency values in the text and figures are derived from these within neuron comparisons. Synchronized activity is referred to as network events to clarify activity measures taken from calcium imaging data. Similar rhythmic activity measured with field and patch-clamp recordings from individual cells are referred to as network bursts throughout the manuscript.

### Field Recordings and Analysis

dMEC, sMEC, NeoC (perirhinal cortex), and the different hippocampal areas were visually identified with an Olympus microscope (4x lens) using oblique contrast. Field electrodes (chloride-coated silver electrodes inserted into 2–3 MΩ borosilicate glass pipettes filled with ACSF) were lowered into the regions of interest using micromanipulators (Luigs-Neuman, Ratingen), and placed at regions with good signal-to-noise readings. Field potentials were acquired with Multiclamp 400B amplifiers (Molecular Devices) using Clampex 10.2 (MDS Analytical Technologies) at an acquisition rate of 10 kHz.

Analysis was carried out using custom-made Matlab^®^ (Mathworks) software. First, signals were high-pass filtered at 1 Hz to eliminate any slow frequency drift of the signal. Then, a threshold of 5.5 times SD was applied to detect field potentials. For the dMEC-sMEC recordings customized thresholds (between 4.5 and 12 times SD) were used to achieve the optimal detection rates. Spikes were grouped as one burst if they were preceded by another spike within 3 s. The first spike of a burst was defined as the burst onset and used to calculate event frequency. Given the long duration of network bursts, a synchronous event between different regions and sMEC was defined as the occurrence of a burst in the region of interest within a time-window of 2.5 s preceding or following an sMEC event. The proportion of synchrony between different regions relative to sMEC was then calculated.

### Patch-Clamp Recordings

**FIGURE 2:**
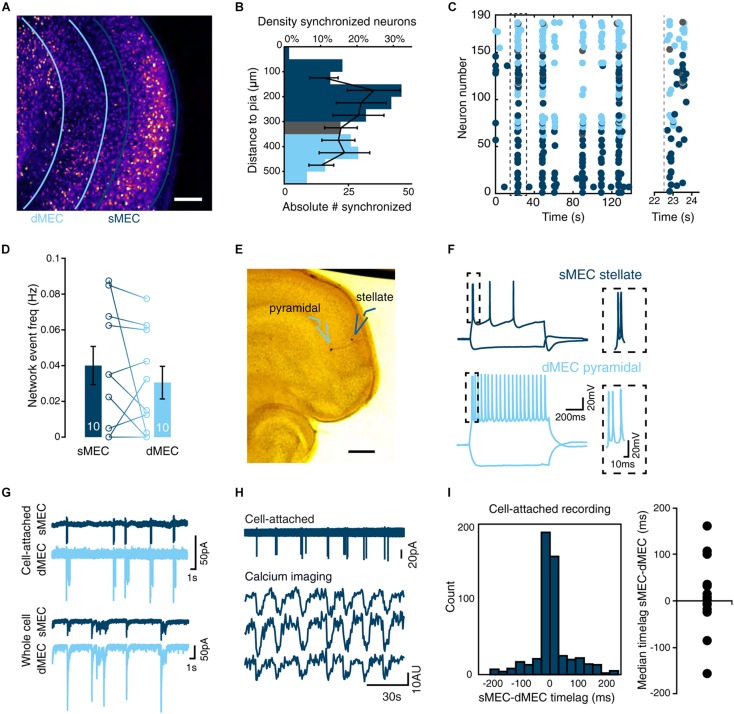
Synchrony between deep and superficial layer networks in immature MEC. **(A)** Two-photon image of entire intact MEC loaded with Fura2-AM calcium dye. Scale bar 100 μm. **(B)** Density of synchronized neurons (black lines, mean ± SEM) is not significantly different between deep (light-blue) and superficial layers (dark-blue) and intermediate (gray) layers [one-way ANOVA, *F*(7,69) = 0.76, *p* = 0.622]. Absolute number of synchronized neurons per distance bin indicated as bars. *N* is 8 slices. **(C)** Left: Representative raster plot of deep, superficial and intermediate layer MEC activity showing correlated synchrony across layers. Right: magnified view of the first network event indicated on the left by the dashed black box. Gray dashed line indicates onset of activity. **(D)** Quantification reveals no significant difference in frequency of synchronized network activity between deep and superficial networks [paired *t*(9) = 0.98, *p* = 0.353]. *N* shown in bars. **(E)** Representative biocytin-filled spiny stellate and deep layer pyramidal neuron taken from paired recordings in deep MEC (dMEC) and sMEC (scale bar 250 μm). **(F)** Spike-profile of layer II stellate cell (top) and deep pyramidal neuron. **(G)** Example of cell-attached (*n* = 14 pairs) and whole-cell recordings (*n* = 6 pairs) showing synchrony of spike rates and synaptic inputs. **(H)** Example trace of a cell-attached recording in sMEC and simultaneously recorded calcium imaging traces from three neighboring neurons. **(I)** Histogram of the observed time lag for event onset using high temporal resolution cell-attached recordings (*n* = 14 pairs) show correlated synchrony between deep and superficial layers (left) but no directionality (right, Wilcoxon signed rank test *W* = 64, *p* = 0.52) in immature MEC. Bar graphs represent the mean ± SEM. *N* shown in bars as the number of slices.

For patch-clamp recordings, the same recording apparatus was used as for field recordings (see above), but the microscope was equipped with a 60x lens. Borosilicate pipettes with a resistance of ∼10 MΩ were filled with intracellular solution containing (in mM): 148 Kgluconate, 1 KCl, 10 Hepes, 4 Mg-ATP, 4 K_2_-phosphocreatine, 0.4 GTP, and 0.2% biocytin, adjusted with KOH to pH 7.3. For cell-attached recordings in Figure 2 (2–10 min), active cells of interest were selected in dMEC and sMEC and recorded simultaneously in GΩ-resistance seal configurations. After up to 10 min of continuous voltage-clamp recordings, whole-cell access was made if possible and step protocols were acquired in whole-cell current-clamp mode to electrophysiologically identify neuron type and to fill neurons with biocytin for *post hoc* anatomical confirmation. Stellate cells were electrophysiologically identified based on their characteristic sag and prominent rebound amplitudes often evoking a rebound action potential that pyramidal cells do not have (Canto and Witter, 2012). To measure synaptic inputs of identified neurons in some cases up to 10 min voltage-Clamp, whole-cell recordings were acquired. After recordings, slices were fixed in 4% paraformaldehyde (PFA) for at least 1 week before staining.

**FIGURE 3:**
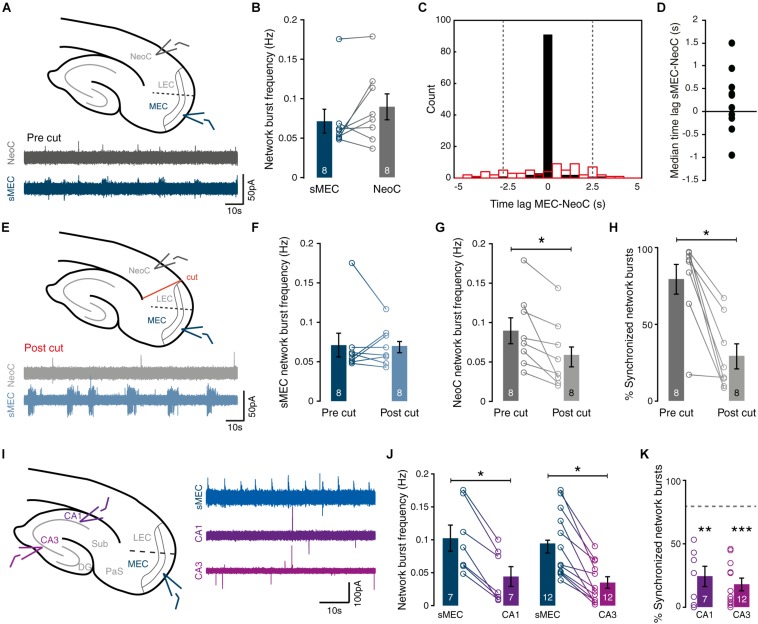
Spontaneous network activity in immature MEC synchronizes network activity in neocortex. **(A)** Top: Schematic view of field recordings. Bottom: Example traces of simultaneous recordings from sMEC (blue) and superficial neocortex (NeoC, gray). **(B)** Network burst frequency is similar between sMEC and NeoC [paired *t*(7) = 1.58, *p* = 0.157]. **(C)** Representative time-lag histogram for onset times of network bursts between sMEC and NeoC pre- (black bars) and post-lesion (red open bars, see **E**). All bursts with a time lag within ± 2.5 s (between gray dotted lines) are considered synchronized. **(D)** No reliable time-lag [one-sample *t*(9) = 1.06, *p* = 0.317] indicating no directionality of activity between sMEC and NeoC. **(E)** Top: Schematic view of field recordings and lesion site. Bottom: Example traces of simultaneous recordings from sMEC (blue) and superficial neocortex (NeoC, gray) after lesion (“post cut”). **(F)** No change in the frequency of sMEC network bursts following lesion [paired *t*(7) = 0.10, *p* = 0.923]. **(G)** Lesion decreased the frequency of NeoC network bursts [paired *t*(7) = 3.19, *p* = 0.015]. **(H)** Decrease in synchronized bursts between sMEC and NeoC after lesion [paired *t*(7) = 5.10, *p* = 0.001]. **(I)** Left: field recording sites (CA1, CA3, sMEC). Right: example traces of simultaneous recordings. **(J)** Network burst frequencies are lower in CA1 and CA3 than in sMEC [CA1: paired *t*(6) = 6.95, *p* < 0.001. CA3: paired *t*(11) = 6.12, *p* < 0.001]. **(K)** Levels of synchrony between CA1-sMEC and CA3-sMEC are significantly weaker than sMEC-NeoC (gray dashed line) [CA1-sMEC vs. NeoC-sMEC: *t*(15) = 3.87, *p* = 0.002; CA3-sMEC vs. NeoC-sMEC: *t*(20) = 5.72, *p* < 0.001]. Bar graphs represent the mean ± SEM. *N* shown in bars as the number of slices. *p < 0.05; **p < 0.01; ***p < 0.001.

Cell-attached data were analyzed using Matlab^®^ (Mathworks) scripts adapted from the field recording analysis. Due to stable baselines, no filtering was applied. Customized thresholds for action-potential (AP) detection ranged from 2 to 40 times SD. APs were grouped to be part of one network burst if they were preceded by another spike within a time-window of 1.5 s. As a measure of synchrony, the proportion of bursts in sMEC that are followed or preceded within 1 s from time of burst onset in the dMEC was calculated. For anatomical identification, slices were stained for biocytin using a modified avidin-biotin-peroxidase method. Note that staining of the entire dendritic tree was limited caused by the high input resistance due to the 10 MΩ pipettes.

### DiI Tracing

To trace the entorhinal innervation of hippocampus *ex vivo*, horizontal slices were prepared as described above. Slices were allowed to recover in ACSF for one hour and fixed in 4% PFA in phosphate buffered saline for 1 h at room temperature. 1,1′-Dioctadecyl-3,3,3′3′-tetramethylindocarbocyanine perchlorate (Vybrant DiI, Invitrogen) was pressure-injected into the entorhinal cortex. After incubation at 37°C in PBS containing 0.2% ethylenediaminetetraacetic acid (EDTA) and 1% PFA for 1 week, slices were rinsed in PBS. Staining with PBS containing 4′,6-diamidino-2-phenylindole (DAPI, Thermo Fisher Scientific) for 5 min was used to visualize cell bodies and slices were then mounted in Fluoromount. Image stacks were obtained with a Leica TCS-SP8 confocal using a 20x air objective (NA 0.5) with a resolution of 1.51 μm by 1.51 μm by 2 μm (*xyz*) per voxel.

### Statistics

**FIGURE 4:**
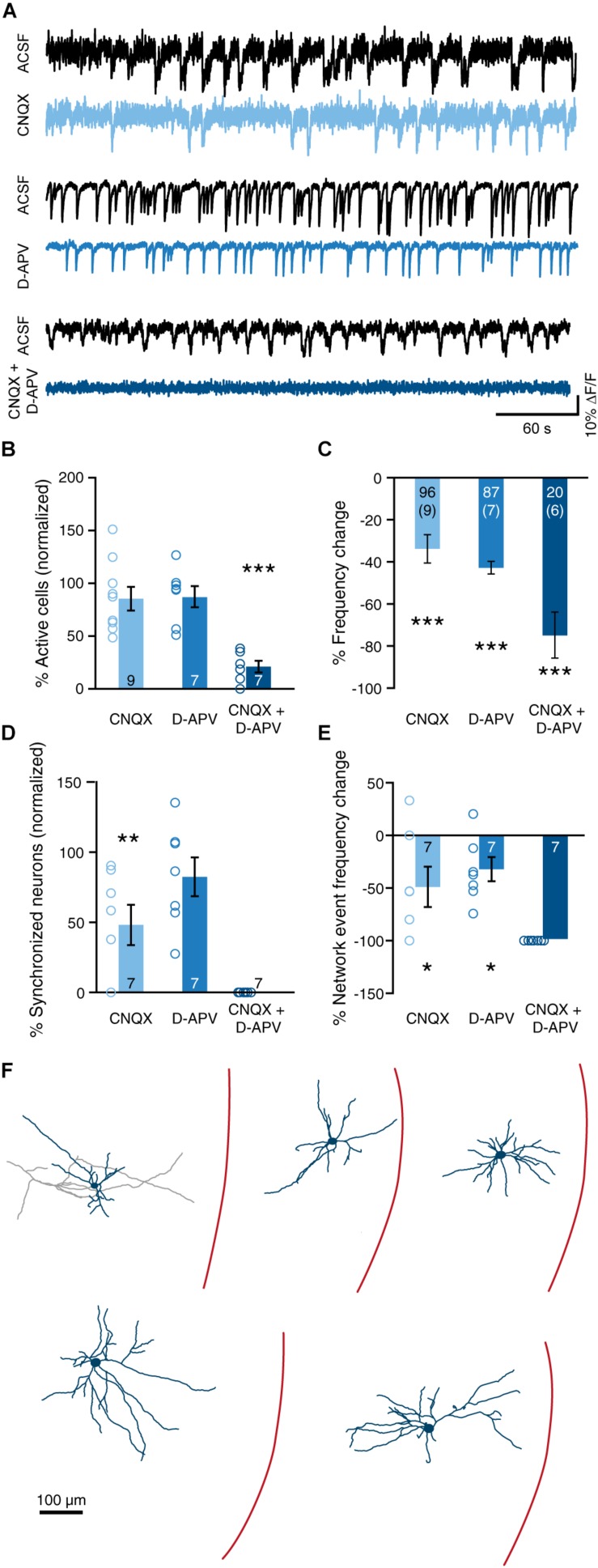
Ionotropic glutamatergic signaling drives spontaneous synchronized network activity in immature sMEC. **(A)** Example traces of individual neurons before (ACSF, black) and after inhibition of AMPA (CNQX, top) receptors, NMDA (D-APV, middle) receptors, and both simultaneously (CNQX + D APV, bottom). **(B)** Blockade of AMPA or NMDA receptors does not significantly affect the proportion of active cells [CNQX: paired *t*(8) = 1.88, *p* = 0.096; D-APV: paired *t*(6) = 1.20, *p* = 0.277]. Simultaneous inhibition of both receptors decreases the proportion of active cells [CNQX + D-APV: paired *t*(6) = 8.70, *p* < 0.001]. **(C)** Blockade of AMPA and/or NMDA receptors decreased overall frequency of activity in permanently active cells [CNQX: paired *t*(95) = 5.11, *p* < 0.001; D-APV: paired *t*(86) = 14.53, *p* < 0.001; CNQX + D-APV: paired *t*(19) = 6.93, *p* < 0.001]. *N* shown in bars, number of slices shown in brackets. Note that one slice in the CNQX + D-APV group did not contain any cells that were active in both the ACSF and drug condition. **(D)** Inhibition of AMPA, but not NMDA, receptors decreased the proportion of clustered cells [CNQX: paired *t*(6) = 4.39, *p* = 0.005; D-APV: paired *t*(6) = 1.22, *p* = 0.268]. Data shown as percentage of cells that were clustered in the ACSF condition. Note that in two slices in the CNQX group, no network events were present in the ACSF condition, and that these slices were excluded from the network activity analysis. Simultaneous inhibition of both AMPA and NMDA receptors abolished all network activity. **(E)** Blockade of either AMPA or NMDA receptors caused a reduction in the frequency of network events [CNQX: paired *t*(6) = 2.48, *p* = 0.0481; D-APV: paired *t*(6) = 3.26, *p* = 0.017]. As CNQX + D-APV abolished all network events, the reduction in frequency was 100% in all slices. **(F)** Reconstructions of Alexa-594-stained intrinsically active neurons. Bar graphs represent the mean ± SEM. *N* shown in bars as either number of slices or “cells (slices).” *p < 0.05; **p < 0.01; ***p < 0.001.

**FIGURE 5:**
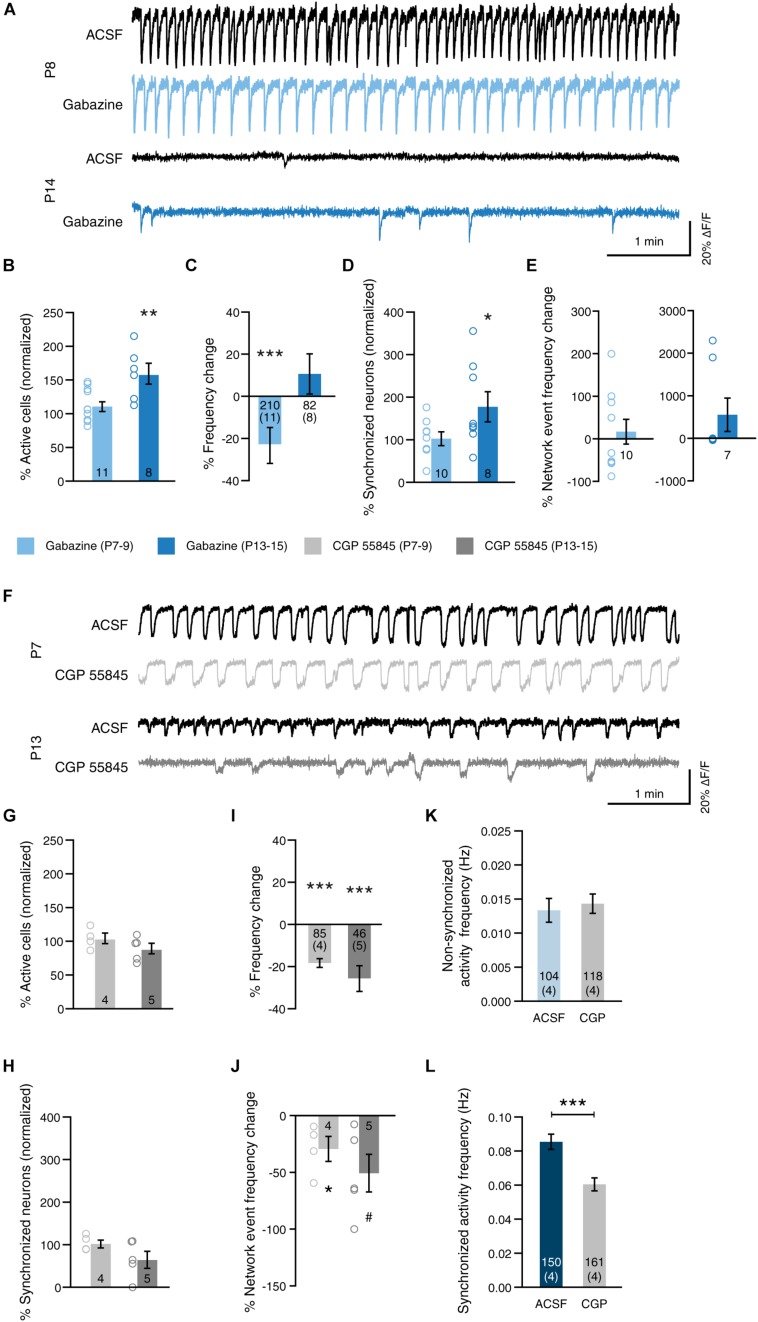
GABA modulates network activity in a receptor-type-dependent manner. **(A)** Example traces before (ACSF, black) and after blockade of GABA-A receptors (Gabazine, blue) at P8 and at P14. **(B)** Blockade of GABA-A receptors at 2 weeks (P13–15) increased the proportion of active cells [gabazine 2 weeks: paired *t*(7) = 4.39, *p* = 0.003]. Blocking GABA-A receptors at 1 week (P7–9), had no significant effect [gabazine 1 week: paired *t*(10) = 1.08, *p* = 0.307]. **(C)** Blockade of GABA-A signaling reduced the frequency of permanently active cells at 1 week, but not at 2 weeks [gabazine 1 week: paired *t*(210) = 2.74, *p* = 0.007; gabazine 2 weeks: paired *t*(82) = 1.17, *p* = 0.244]. **(D)** Blockade of GABA-A receptors at 2 weeks increased the proportion of cells that participate in network events [gabazine 1 week: paired *t*(9) = 1.85, *p* = 0.097; gabazine 2 weeks: paired *t*(7) = 2.44, *p* = 0.045]. Data shown as percentage of cells that were clustered in the ACSF condition. Note that in one slice of the gabazine 1 week group, no network events were present in the ACSF condition, and that this slice was excluded from the network activity analysis. *N* shown in bars. **(E)** The frequency of network events was not affected by inhibition of GABA-A receptors [gabazine 1 week: paired *t*(9) = 1.46, *p* = 0.178; gabazine 2 weeks: Wilcoxon signed rank *W* = 0, *p* = 1.0]. *N* as in **(D)**. **(F)** Example traces before (ACSF, black) and after blockade of GABA-B receptors (CGP 55845, gray) at P8 and at P14. **(G)** Blockade of GABA-B receptors at either age had no significant effect on the proportion of active cells [CGP 55845 1 week: paired *t*(3) = 0.21, *p* = 0.847; CGP 55845 2 weeks: paired *t*(4) = 1.67, *p* = 0.170]. **(H)** Blockade of GABA-B receptors did not the proportion of cells that participate in network events [CGP 55845 1 week: paired *t*(3) = 0.35, *p* = 0.751; CGP 55845 2 weeks: paired *t*(4) = 1.42, *p* = 0.228]. Data shown as percentage of cells that were clustered in the ACSF condition. **(I)** Blockade of GABA-B signaling reduced the frequency of permanently active cells at both ages [CGP 55845 1 week: paired *t*(85) = 9.05, *p* < 0.001; CGP 55845 2 weeks: paired *t*(46) = 4.26, *p* < 0.001]. **(J)** The frequency of network events was reduced by inhibition of GABA-B receptors at 1 week [CGP 55845 1 week: paired *t*(3) = 3.41, *p* = 0.042; CGP 55845 2 weeks: paired *t*(4) = 2.40, *p* = 0.074]. **(K)** The frequency of activity that is not part of network events is unchanged by blocking GABA-B receptors at 1 week (Mann–Whitney *U* = 5996, *p* = 0.769). **(L)** The frequency of activity that is part of network events is reduced by GABA-B receptor blockade (Mann–Whitney *U* = 5644, *p* < 0.001). Bar graphs represent the mean ± SEM. *N* shown in bars as either number of slices or “cells (slices).”

Statistical tests used are indicated in the figure legends for individual experiments. In general, to compare two conditions within the same experiment, a paired *t*-test was used unless data were not normally distributed and then a Wilcoxon Signed Rank test was used. To compare means from different experiments independent *t*-tests were performed, or Mann–Whitney *U*-tests when data were not normally distributed. Multiple means were compared using one-way ANOVAs. *Post hoc* analysis was carried out using Bonferroni-corrected *t*-tests. Reported is the abbreviation of test statistic with the degrees of freedom in brackets equaling the value of the test statistic. Differences were defined as significant if *p*-values were smaller than 0.05. In figures, ^∗^ indicates significance (*p* < 0.05), # indicates a trend (*p* < 0.1). All statistics were performed on slice level except the % frequency change in Figures 4, 5, and the activity frequencies in Figure 5. Data regarding glutamate and GABA receptor blockers are shown in figures as percentages of baseline values or percentage changes relative to baseline values, in order to make effect sizes more readily comparable. However, all statistical tests on these data are performed on the raw values (see Table 1).

**TABLE 1:** Non-normalized values for all data shown in Figures 4, 5.

Measurement	Pre (mean ± SEM)	Post (mean ± SEM)
**CNQX**		
Fraction active cells	0.58 ± 0.08	0.48 ± 0.07
Fraction synchronized cells	0.41 ± 0.1	0.26 ± 0.11
Overall event frequency	0.07 ± 0.01	0.04 ± 0.01
Network event frequency	0.03 ± 0.01	0.01 ± 0.01
**D-APV**		
Fraction active cells	0.54 ± 0.05	0.47 ± 0.06
Fraction synchronized cells	0.46 ± 0.04	0.38 ± 0.07
Overall event frequency	0.12 ± 0	0.07 ± 0
Network event frequency	0.12 ± 0.01	0.08 ± 0.01
**CNQX + D-APV**		
Fraction active cells	0.54 ± 0.05	0.13 ± 0.03
Fraction synchronized cells	0.46 ± 0.04	0 ± 0
Overall event frequency	0.13 ± 0.01	0.03 ± 0.01
Network event frequency	0.12 ± 0.01	0 ± 0
**GABAzine (P7-9)**		
Fraction active cells	0.56 ± 0.07	0.6 ± 0.05
Fraction synchronized cells	0.25 ± 0.04	0.39 ± 0.07
Overall event frequency	0.03 ± 0.003	0.02 ± 0.002
Network event frequency	0.04 ± 0.01	0.02 ± 0.004
**GABAzine (P13-15)**		
Fraction active cells	0.26 ± 0.06	0.47 ± 0.09
Fraction synchronized cells	0.33 ± 0.05	0.49 ± 0.06
Overall event frequency	0.06 ± 0.01	0.07 ± 0.01
Network event frequency	0.06 ± 0.02	0.06 ± 0.01
**CGP 55845 (7-9)**		
Fraction active cells	0.61 ± 0.11	0.62 ± 0.08
Fraction synchronized cells	0.5 ± 0.12	0.51 ± 0.11
Overall event frequency	0.1 ± 0.01	0.08 ± 0.005
Network event frequency	0.1 ± 0.03	0.07 ± 0.03
**CGP 55845 (P13-15)**		
Fraction active cells	0.43 ± 0.09	0.38 ± 0.08
Fraction synchronized cells	0.34 ± 0.08	0.28 ± 0.1
Overall event frequency	0.09 ± 0.01	0.07 ± 0.01
Network event frequency	0.07 ± 0.02	0.04 ± 0.02

*Note that the CNQX + D-APV experiment was performed in the same slices as the D-APV experiment, and therefore baseline values are the identical.*

## Results

### Entorhinal Cortex Shows Robust SSNA During the Second Postnatal Week

To unravel the mechanisms underlying SSNA, we utilized an *in vitro* slice live-imaging approach. Using multiphoton calcium imaging with single cell resolution, we found that networks in the sMEC consisted of silent and active cells, of which the majority of the latter were synchronously active (Figure 1A). To establish the developmental time-course of SSNA, we mapped spontaneous network events during the second postnatal week, which precedes the onset of spatially tuned neuronal firing in MEC (Langston et al., 2010; Wills and Cacucci, 2014). In each neuron, somatic calcium events reflecting suprathreshold activity were binarized and their synchronization across the network was calculated (Figures 1B–D, see section “Materials and Methods”). SSNA peaked at P10/11, exhibiting the highest proportion of active neurons (Figure 1C, light-blue bars, 40.1 ± 4.9% cells), and significantly decreased toward P14/15 (7 ± 2% cells, Figure 1C, light-blue bars). Within the active network, the fraction of active neurons that were synchronized followed a similar profile, peaking at P10/11 (Figure 1C, dark-blue bars, 31 ± 4% cells) and decreasing by P14/15 (Figure 1C, dark-blue bars, 2 ± 1% cells). Frequency of synchronized events across the network (“network events”—see section “Materials and Methods” for definition), also peaked at P10/11 (Figure 1D, 0.050 ± 0.013 Hz) and virtually disappeared by P14/15 (Figure 1D, 0.002 ± 0.001 Hz). In summary, SSNA in MEC peaks at P10/11. Unless otherwise stated, all data in the following paragraphs are reported from peak activity ages P8-11 *in vitro*.

### Time-Locked Network Synchrony Across Layers of MEC

Spontaneous synchronized network activity in entorhinal cortex during the first postnatal week is led by activity in layer III (Namiki et al., 2013). We used low-magnification calcium imaging of entorhinal cortex in P10/11 slices to determine whether this holds true during the second postnatal week. Deep (dMEC, light-blue) and superficial (sMEC, dark-blue) MEC was defined prior to event analysis based on cytoarchitecture (Figure 2A). Synchronized neurons were found in both dMEC and sMEC, with no difference in density between layers or relative to pial distance (Figure 2B). Network events in dMEC and sMEC were synchronized across individual neurons (Figure 2C), with a similar frequency (Figure 2D, 0.04 ± 0.01 Hz vs. 0.03 ± 0.01 Hz).

To investigate the temporal relationship between dMEC and sMEC with millisecond resolution, we used paired cell-attached recordings of sMEC stellate cells and dMEC pyramidal neurons (Figure 2E). We measured infrequent periods of activity during which several spikes occurred, which we termed “network bursts” (Figure 2G). Simultaneous cell-attached recording and calcium imaging showed that these network bursts coincided with network events (Figure 2H). At the end of each experiment, we switched to whole-cell configuration for subsequent electrophysiological cell-type identification (Figures 2E–H). As with network events recorded by calcium imaging, network bursts were highly synchronized (Figure 2G; cell-attached recordings: 76 ± 6%, *n* = 14, data not shown). There was no significant time-lag between cell pairs or layers, providing no evidence that superficial layers were driving spontaneous superficial network activity (Figure 2I, cell-attached median time lag: 6 ms IQR: -0.14 to + 0.36 ms). Finally, paired whole-cell voltage-clamp recordings from electrophysiologically identified stellate cells in sMEC and dMEC pyramidal neurons showed strikingly similar spontaneous input patterns during network bursts (Figure 2G, lower traces). In conclusion, we did not observe superficial neurons to be the initiators of network events during the second postnatal week.

### Entorhinal Cortex Drives Synchronous Network Activity in Neocortex

Immature entorhinal cortex has been proposed as a cortical “pacemaker” whose intrinsic activity drives neighboring neocortical regions during early postnatal development (Garaschuk et al., 2000; Namiki et al., 2013). Therefore, we next sought to determine whether network events in the entorhinal cortex were synchronized with activity or driving in different brain regions.

To first confirm that sMEC can self-generate its own SSNA during the second postnatal week, we utilized calcium imaging in isolated MEC mini-slice preparations (Supplementary Figure S1A). Frequency of network events dropped upon isolation (Supplementary Figure S1C, right, 0.12 ± 0.02 vs. 0.06 ± 0.01 Hz). However, we observed no significant changes in fractions of active (Supplementary Figure S1B, left, 47 ± 3 vs. 47 ± 5%), synchronized neurons (Supplementary Figure S1B, right, 31 ± 4 vs. 29 ± 6%) or activity levels (Supplementary Figure S1C, left, 0.04 ± 0.009 vs. 0.02 ± 0.006 Hz) indicating that intrinsic synchrony persists, similar to the intact slice preparation.

To test whether EC bursts drive neocortical activity, we used simultaneous field recordings in both sMEC (blue traces) and NeoC (gray traces) to measure spontaneous network bursts (Figure 3A). Neocortical network bursts were highly synchronized with sMEC bursts, with no significant difference in frequency (Figure 3B, sMEC: 0.79 ± 0.2 Hz, NeoC: 0.089 ± 0.2 Hz). We saw no significant time-lag between sMEC and NeoC to indicate a consistent origin of activity and propagation [Figure 3C (black bins), Figure 3D; average median time lag sMEC-NeoC: 0.230 ± 0.218 s].

To directly test if EC paces NeoC activity, we lesioned all interregional connections (Figure 3E). Both sMEC and NeoC displayed rhythmic network bursts following lesioning (Figure 3E, bottom traces), indicating that neocortical activity must be partly generated by a source other than EC. However, neocortical network burst frequency significantly dropped following separation from EC (Figure 3G), with no change in sMEC network burst frequency (Figure 3F). Importantly, sMEC-NeoC synchrony was strongly decreased post-lesion (Figure 3H, pre: 79 ± 10%, post: 29 ± 8%, Figure 3C, red bins), suggesting that neocortical bursts are driven by EC activity.

To test whether SSNA in sMEC could be driven by the immature hippocampus, we made simultaneous field recordings in CA1-sMEC, CA3-sMEC, and CA1-CA3-sMEC (Figure 3I). Frequency of spontaneous activity was significantly higher in sMEC compared to CA1 (Figure 3J, left, 0.1 ± 0.02 vs. 0.04 ± 0.015 Hz), and CA3 (Figure 3J, right, 0.09 ± 0.015 vs. 0.04 ± 0.008 Hz). Furthermore, the proportion of network bursts that were synchronized with sMEC (Figure 3K) was significantly lower for both CA1 (24 ± 8%) and CA3 (18 ± 5%) compared to synchrony observed between sMEC and NeoC (Figure 3K, dotted line). To show that the lack of clear synchrony between MEC and hippocampus is not caused by disturbed connectivity caused by the slicing procedure, we performed DiI tracing of perforant pathway fibers. Although DiI injections were relatively superficial and therefore prone to be affected by slicing, we observed significant innervation of CA1 and dentate gyrus by fibers originating from entorhinal cortex (Supplementary Figure S2). This indicates that entorhinal-hippocampal connectivity in our slice preparations is not affected more than entorhinal-NeoC connectivity, in line with previous results (Xiong et al., 2017).

In summary, network activity during week 2 is highly synchronized between MEC and NeoC and MEC influences the frequency of activity in NeoC. In contrast, there is little synchrony between MEC and hippocampal regions CA1 and CA3.

### Ionotropic Glutamatergic Activity Underlies Immature MEC Synchrony

Spontaneous synchronized network activity has previously been shown to be dependent on glutamatergic transmission (Garaschuk et al., 2000; Allène et al., 2008; Namiki et al., 2013). We sought to gain a better understanding of how glutamatergic transmission through AMPA and NMDA receptors affects activity of individual neurons, and how they are enlisted into network events. AMPA and NMDA receptors were selectively blocked by bath application of 2 μM CNQX and 100 μM D-APV, respectively (Figure 4A and Table 1). Blockade of either receptor did not decrease the percentage of neurons that showed activity, whereas application of both blockers simultaneously caused a large reduction in the number of active neurons (Figure 4B). We then analyzed the frequency of neurons that were active both in the ACSF condition and after drug application. CNQX and D-APV individually caused a reduction in event frequency 34.2 ± 6.7 and 43.2 ± 3.0%, respectively (Figure 4C). Application of CQNX and D-APV simultaneously reduced event frequency by 75.0 ± 10.8% (Figure 4C).

Application of CNQX, but not D-APV, significantly reduced the fraction of synchronized neurons (Figure 4D). Either compound caused a small reduction in the frequency of network events (Figure 4E). In line with previous studies (Sheroziya et al., 2009; Namiki et al., 2013), the combination of CNQX and D-APV completely blocked the occurrence of network events (Figures 4D,E). Thus, SSNAs during the second postnatal week are driven by glutamatergic synaptic transmission, with AMPA receptors in particular playing a large role in attracting cells into network events.

Note that even when blocking both AMPA and NMDA receptors, on average approximately 13% of neurons within a slice continued to show activity. We performed cell-attached recordings of these neurons and filled them with Alexa-594. Reconstructions of these Alexa-594-stained neurons revealed mixed multipolar morphologies among which stellate-like cells (Figure 4F).

### GABAergic Signaling Modulates Network Activity

Spontaneous synchronized network activity in sMEC becomes asynchronous and sparse at the end of the second postnatal week (Figure 2, see also Golshani et al., 2009 for data in NeoC). We hypothesized that increased GABAergic tone and maturation of the GABAergic system could mediate the abolition of SSNA like in NeoC and hippocampus (Garaschuk et al., 2000; Allène et al., 2008; for review: Blankenship and Feller, 2009). Thus, to determine the role of GABA, we tested the blockade of both GABA-A and GABA-B receptors during synchronous activity at the start of the second postnatal week (P7–9), and during asynchronous sparse activity at P13–15 (Figures 5A,F and Table 1).

Blockade of GABA-A receptors by bath application of 10 μM gabazine increased the number of active neurons at the end of the second postnatal week, but not the end of week 1 (Figures 5A,B, week 1: 112.3 ± 7.1% of baseline, week 2: 159.3 ± 15.6% of baseline). Interestingly, when looking at neurons that are active both in the baseline condition and after wash-in of the drug, blockade of GABA-ARs reduced the frequency of those neurons after 1 week, but not after 2 weeks (Figure 5C, week 1: -23.3 ± 8.5% compared to ACSF, week 2: 11.22 ± 9.6% compared to ACSF). At 1 week, blockade of GABA-ARs did not significantly affect either, the fraction of synchronized neurons participating in network events, or network event frequency (Figures 5D,E, light-blue, synchronized neurons: 5.6 ± 16.1% compared to ACSF, network event frequency: 119.9 ± 29.1% of baseline). At 2 weeks, on the other hand, GABA-A receptor blockade significantly increased the number of synchronized cells that participate in network events (Figure 5D, dark-blue, 80.4 ± 35.3% compared to ACSF).

In juvenile MEC (third postnatal week), metabotropic GABA-B receptors play a role in terminating persistent network activity of layer III pyramidal neurons (Mann et al., 2009). In contrast to GABA-A receptor blockade, GABA-B receptor blockade by 4 μM CGP 55845 did not significantly increase the number of active nor synchronously active neurons (Figures 5F–H, active neurons: week 1, 104.4 ± 7.8%, week 2, 89.3 ± 7.9% of baseline; synchronized neurons: week 1, + 4.7 ± 9.1%, week 2, -32.8 ± 20.0% compared to ACSF). However, inhibition of GABA-B receptors decreased the frequency of activity in general (Figure 5I, week 1: -18.9 ± 2.1%; week 2: -26.2 ± 6.2% compared to ACSF), and the frequency of synchronous network events (Figure 5J, week 1: 70.8 ± 11.0%; week 2: 48.2 ± 16.6% of baseline). Because we have found that most neuronal activity in our slice preparations is part of network events (see Figure 1), we hypothesized that GABA-B primarily affects event frequency, which in turn affects the readout of frequency in general. To test this, we separated individual calcium events at 1 week into those that were part of a network event (“synchronized activity”) and those that were not part of a network event (“non-synchronized activity”). We found that the frequency of non-synchronized activity was not significantly altered by GABA-B receptor blockade (Figure 5K, ACSF: median 0.010 Hz, IQR 0.005–0.018 Hz; CGP 55845: median 0.010 Hz, IQR 0.004–0.023 Hz). In contrast, the frequency of synchronized activity was reduced by blocking GABA-B receptors (Figure 5L, ACSF: median 0.078 Hz, IQR 0.059–0.080 Hz; CGP 55845: median 0.050 Hz, IQR 0.045–0.055 Hz). We therefore conclude that GABA-B receptors primarily affect synchronous network activity, rather than activity in general, in MEC at this stage of development.

Thus, GABA-A and GABA-B have opposing effects on activity and synchrony in developing MEC. Importantly, GABA-A and GABA-B receptors do not drive SSNA but significantly modulate network activity levels overall by P13-15 and regulate activity in comparable patterns to those reported from the third postnatal week onward.

## Discussion

In this study, we asked whether the characteristics and underlying mechanisms of SSNA during the second postnatal week resemble the activity during the first week or whether there are distinct features during this period. We found some characteristics and underlying mechanisms that are similar to week 1. In particular, we confirm that MEC drives synchrony in NeoC and that SSNA in MEC is dependent on iGluRs. In contrast, we show that SSNA in week 2 displays distinct characteristics; specifically, we find a lack of correlation with hippocampal activity and differential effects of GABA-A and GABA-B receptor activity on the network. These results suggest different or additional functions of MEC network activity within the hippocampal-entorhinal circuitry with increasing maturation.

Using a calcium imaging approach with single cell resolution across layers, we find that SSNA in sMEC peaks at P10–11, in line with previous studies that reported similar activity using field potential recordings in specific layers (Jones and Heinemann, 1989; Sheroziya et al., 2009). In contrast, Garaschuk et al. (2000) report that network bursts vanish in entorhinal cortex after P6. The peak frequency we measure (0.06 Hz at P10/11) is similar to the burst frequencies reported in earlier studies (Jones and Heinemann, 1989; Sheroziya et al., 2009). However, entorhinal network activity during the first postnatal week reported by Garaschuk et al. (2000) was markedly slower (median frequency 0.004 Hz) and only increased to similar frequencies right before activity stopped (numbers not reported). Thus, our results on SSNA during the second postnatal week in MEC are in line with most previous studies.

### Synchrony of Network Activity Within MEC and Across Brain Regions

Our data show that SSNA in MEC during the second postnatal week is highly temporally correlated between superficial and deep layers of MEC. This is in contrast to lateral EC during the first postnatal week, where cells in layer III reliably precede those in deeper layers (Namiki et al., 2013). One possibility to explain this difference is that SSNA becomes more synchronous across layers as it spreads from the initiation site. Thus, while neurons in layer III precede those in deeper layers in LEC near the initiation site, this asynchrony is no longer present when the network burst reaches the MEC. Alternatively, assemblies of neurons that are synchronously active may grow in size with age, so that by the second postnatal week network events span multiple layers. A similar pattern has been observed for assemblies of GABAergic neurons in barrel cortex, which increased in size over the course of several days (Mòdol et al., 2020). In line with this, P10–11 is also the age range at which the largest proportion of neurons participates in network event, after which activity becomes more sparsely distributed.

Interestingly, this coincides with the decorrelation of activity in the barrel cortex, a process that is unaffected by deprivation of whisker input (Golshani et al., 2009). We find that, similar to the first postnatal week (Namiki et al., 2013), SSNA in the EC during the second postnatal week sets the pace for network activity in the NeoC. Thus, our results further substantiate the role of the MEC as a developmental hub (Garaschuk et al., 2000; Mòdol et al., 2017). Therefore, we hypothesize that sparsification of activity in the EC toward the end of the second postnatal week could act as a signal for desynchronization of activity in the NeoC.

In contrast to the highly synchronized activity between NeoC and MEC, we do not observe a high degree of synchronized activity between the MEC and the hippocampus during the second postnatal week. This is contrary to what is observed during the first postnatal week *in vivo* (Valeeva et al., 2019) and *in vitro* (Namiki et al., 2013). SSNA in hippocampus drops off earlier during development than that in EC, around P8-10 (Ben-Ari et al., 1989; Garaschuk et al., 1998). This might well be because hippocampal network activity relies on depolarizing actions of GABA (Ben-Ari et al., 2007), and GABA becomes inhibitory in the hippocampus around that time (Ben-Ari et al., 2007; Murata and Colonnese, 2019). Indeed, network burst frequency as measured by field potential recordings was consistently lower in hippocampus than in EC. Thus, while EC drives hippocampal network activity during the first postnatal week, synchrony between these brain regions disappears as activity in the hippocampus is desynchronized during the second postnatal week.

### Synaptic Mechanisms of Network Activity

We confirm that iGluR activation is the synaptic mechanism that underlies immature MEC SSNA also in week 2 (Jones and Heinemann, 1989; Garaschuk et al., 2000; Sheroziya et al., 2009; Namiki et al., 2013). Interestingly, there are some cells that remain active after iGluR blockade, similar to what (Namiki et al., 2013) report during week 1. At least some of those cells resemble stellate cell morphologies. It is possible that these cells generate spontaneous activity that initiates network events in MEC. Interestingly, from the third postnatal week on, stellate cells in MEC drive the maturation of other cell types in both EC and hippocampus (Donato et al., 2017). We thus hypothesize that stellate cells may be involved in generating MEC network activity during the second postnatal week, in addition to their role in maturation of the hippocampal-EC circuit during later development.

Where GABAergic signaling drives SSNA in hippocampus (Garaschuk et al., 1998) and is suggested to terminate SSNA in other brain regions (for review: Blankenship and Feller, 2009), it plays a more subtle role in network activity in the entorhinal cortex. At the end of the first postnatal week, GABAergic neurons in MEC may be hub neurons that can single-handedly influence network activity (Mòdol et al., 2017). In our data, GABA-A signaling does not influence the frequency of network events or the participation of individual cells in those events. This could be explained by individual (hub) neurons that can increase or decrease network event frequency (Luccioli et al., 2018). Thus, blocking all GABA-A signaling may cancel out effects that individual (hub) neurons have on the network.

Additionally, we find that the effect of GABAergic transmission on network event frequency can be mediated by GABA-B receptors, as blocking GABA-B receptors decreases the frequency of network events. This seems counterintuitive, as the main function of GABA-B receptors is to inhibit neurotransmitter release through inhibition of N-type and P/Q-type calcium channels and activation of inwardly rectifying potassium channels (Pinard et al., 2010). Indeed, activation of GABA-B receptors during the third postnatal week decreases the excitability of stellate cells in the EC (Deng et al., 2009).

Notably, the distribution and action of GABA-B receptors may not be homogenous throughout the network. Research in the prefrontal cortex has shown that presynaptic GABA-B activation causes short-term depression of inhibitory inputs, and that inputs from somatostatin (SST)-expressing interneurons (SST-INs) are more strongly affected than those of parvalbumin (PV)-expressing interneurons (Liu et al., 2017). Hub cells that strongly influence SSNA tend to be SST-INs (Mòdol et al., 2017). Therefore, the effect we find of GABA-B signaling on network activity might be mediated through inputs from this cell type. However, the exact mechanism through which GABA-B activation influences SSNA, and whether this process involves SST-INs remains to be determined.

The effect of GABAergic signaling on activity in the network changes with age. We find that blockade of GABA-A receptors decreases the activity of neurons at the end of the first postnatal week, indicating a depolarizing effect of GABA at this age (Ben-Ari et al., 2007). However, we did not observe GABA-driven cortical giant depolarizing potentials (cGDPs, Allène et al., 2008), indicating that these are restricted to the NeoC at this age. In contrast, blocking GABA-A receptors at the end of the second postnatal week increases both the number of active cells in the slice and the proportion of cells participating in the network. Thus, there seems to be a proportion of neurons that is integrated in the network but fails to participate in network events due to their being silenced by GABAergic inhibition. Hence, increased inhibitory GABAergic drive toward the end of the second postnatal week contributes to sparsification of the network, similar to what was shown in NeoC before (Garaschuk et al., 2000). However, in contrast to what has been reported before in NeoC (Garaschuk et al., 2000), blockade of GABA-A receptors does not reinitialize SSNA at the end of the second postnatal week. We can therefore conclude that while GABA-A dependent inhibition contributes to sparsification of SSNA, it is not the mechanism through which network activity terminates during development. This may instead be due to more cell-intrinsic mechanisms, such as a developmental decrease in input resistance and therefore excitability (Burton et al., 2008). Thus, while we have made strides to elucidate the synaptic mechanisms underlying SSNA, we have yet to determine the precise origin of this characteristic activity and the cause for its termination during development.

## Data Availability Statement

The datasets generated for this study are available on request to the corresponding author.

## Ethics Statement

The animal study was reviewed and approved by the Animal Ethics Committee (Dierexperimentele Commissie DEC) of the Vrije Universiteit Amsterdam.

## Author Contributions

JD and TK performed and analyzed the experiments. TK, JD, and RM wrote the manuscript. JH wrote the scripts for the calcium data analysis. JD wrote the scripts for the analysis of electrophysiological experiments. RM and HM supervised the project.

## Conflict of Interest

The authors declare that the research was conducted in the absence of any commercial or financial relationships that could be construed as a potential conflict of interest.
