# Supplementary material for: Distinct Synchronous Network Activity During the Second Postnatal Week of Medial Entorhinal Cortex Development
Source: Front Cell Neurosci. 2020 Apr 21;14:91. doi: 10.3389/fncel.2020.00091 (PMC7186407; doi:10.3389/fncel.2020.00091)
Supplement: Supplementary file 1 [file Image_1.PDF]

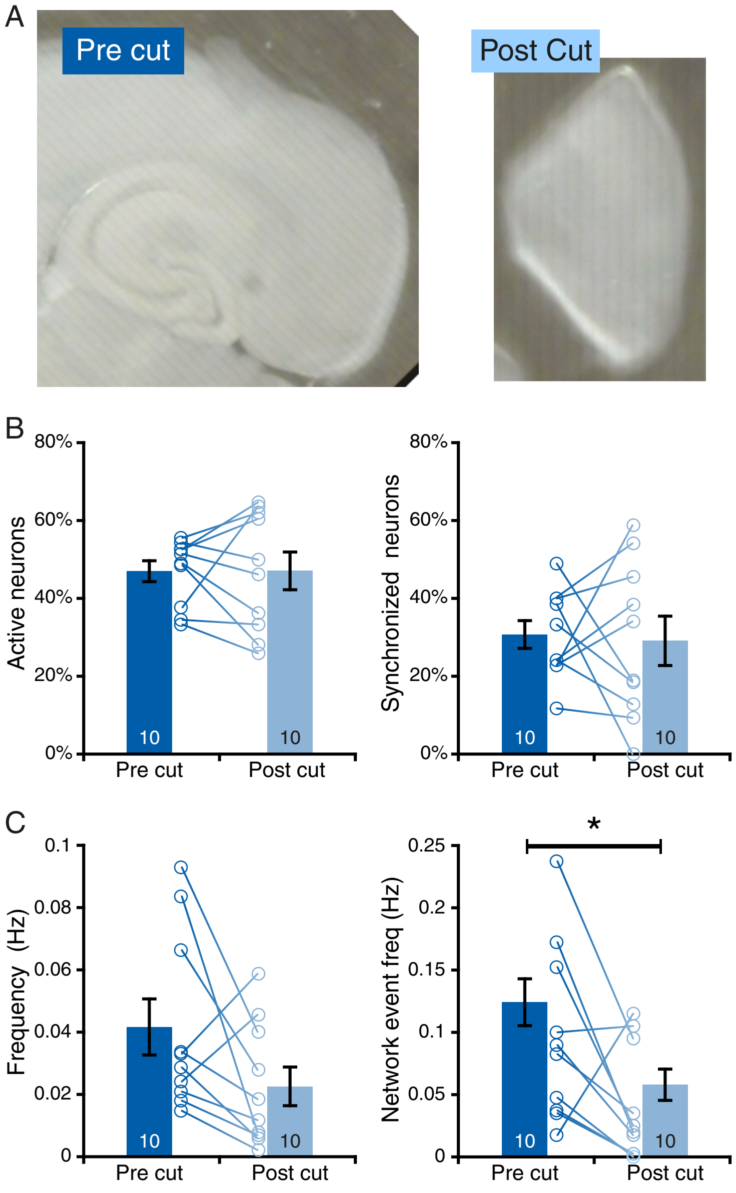

Supplementary Figure 1: Spontaneous synchronized network activity is intrinsic to the developing MEC. (**A**) Horizontal brain slice before and after MEC dissection. (**B**) Lesion caused no change in the number of active (left) or synchronized (right) neurons in sMEC (paired $t(9) = -0.02$, $p = 0.983$ and $t(9) = 0.23$, $p = 0.82$). (**C**) Isolation of MEC from hippocampus, pre- and parasubiculum and neocortex did not cause a significant decrease in frequency of activity (left, paired $t(9) = 1.95$, $p = 0.08$) but decreased the frequency of synchronized events (paired $t(9) = 2.34$, $p < 0.05$). Number of slices tested indicated on individual bars.
