# Supplementary material for: Distinct Synchronous Network Activity During the Second Postnatal Week of Medial Entorhinal Cortex Development
Source: Front Cell Neurosci. 2020 Apr 21;14:91. doi: 10.3389/fncel.2020.00091 (PMC7186407; doi:10.3389/fncel.2020.00091)
Supplement: Supplementary file 2 [file Image_2.PDF]

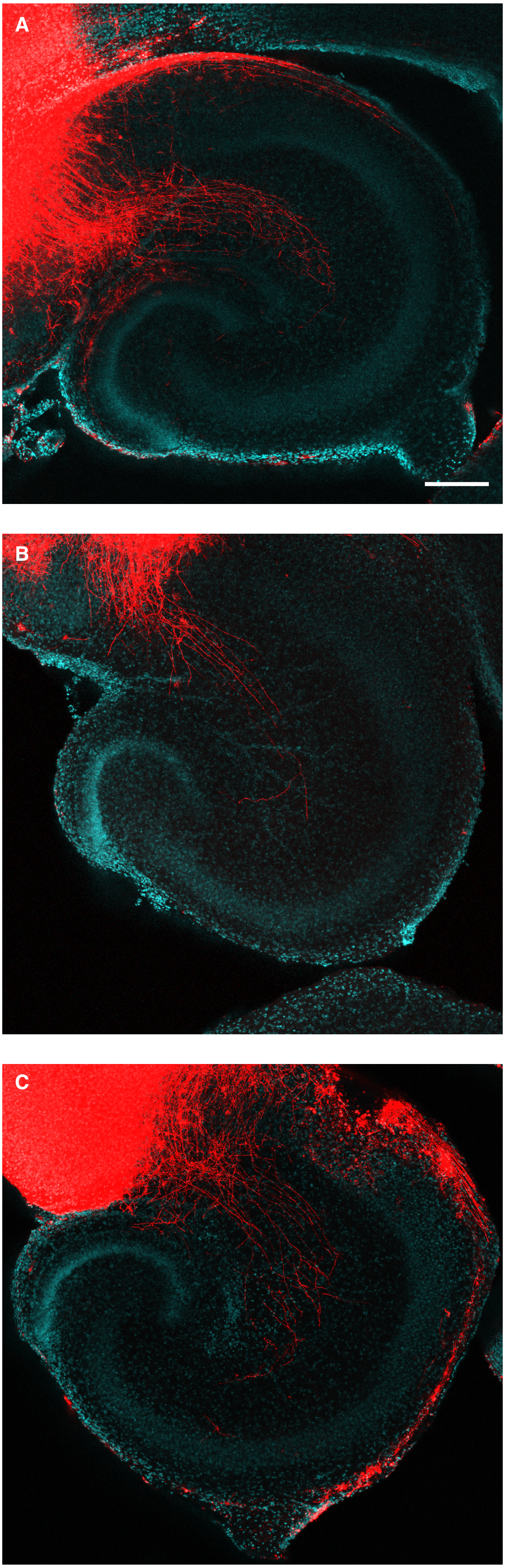

Supplementary Figure 2: Tracing of entorhinal-hippocampal connectivity after slice preparation. 300 µm slices were prepared from three animals in the same manner as for field recordings and then fixed. DiI was injected into the entorhinal cortex after fixation and allowed to diffuse for one week. (**A-C**) Z-projection of DiI signal (red) in one example slice for each animal. In each slice, the top 70 - 80 µm could be imaged. DAPI was used to visualise hippocampal structures (cyan). Scale bar, 200 µm.
